# Activated Carbon Derived from Waste Oil Shale Semi-Coke for Supercapacitor Application

**DOI:** 10.3390/molecules28124804

**Published:** 2023-06-16

**Authors:** Chu’an Xiong, Nan Wang, Mai Feng

**Affiliations:** College of Environmental and Chemical Engineering, Heilongjiang University of Science and Technology, Harbin 150027, China

**Keywords:** oil shale semi-coke, activated carbon, supercapacitors

## Abstract

As fossil fuels gradually deplete, oil shale, one of the world’s largest energy resources, has attracted much attention. Oil shale semi-coke (OSS) is the main byproduct of oil shale pyrolysis, which is produced in large quantities and causes severe environmental pollution. Therefore, there is an urgent need to explore a method suitable for the sustainable and effective utilization of OSS. In this study, OSS was used to prepare activated carbon by microwave-assisted separation and chemical activation, which was then applied in the field of supercapacitors. Raman, XRD, FT-IR, TEM, and nitrogen adsorption–desorption were adopted to characterize activated carbon. The results showed that ACF activated with FeCl_3_-ZnCl_2_/carbon as a precursor has larger specific surface area, suitable pore size, and higher degree of graphitization compared with the materials prepared by other activation methods. The electrochemical properties of several active carbon materials were also evaluated by CV, GCD, and EIS measurements. The specific surface area of ACF is 1478 m^2^ g^−1^, when the current density is 1 A g^−1^, the specific capacitance is 185.0 F g^−1^. After 5000 cycles of testing, the capacitance retention rate was as high as 99.5%, which is expected to provide a new strategy of converting waste products to low-cost activated carbon materials for high-performance supercapacitors.

## 1. Introduction

As one of the largest energy resources in the world, oil shale has attracted an increasing amount of attention, especially with the gradual depletion of coal and oil [1,2]. As the main by-product of oil shale pyrolysis, about 10–30 tons of oil shale semi-coke (OSS) can be produced in each ton of shale oil refining process [3,4]. Because of the low combustion value of OSS, the direct utilization of OSS as fuel is restricted. Stacking or landfilling is still the most common treatment method, which not only occupies a significant amount of land but may also result in soil and groundwater pollution with the release of leachate [5]. There is an urgent need to explore a suitable method for the sustainable and effective utilization of OSS.

OSS is a mixture of carbonaceous residues and minerals [6]. During oil shale retorting in the absence of oxygen at temperatures of 400–520 °C, the composition of the minerals changes negligibly. Therefore, the composition of oil shale, oil shale semi-coke, and oil shale ash are basically the same; the main components of which are SiO_2_, Al_2_O_3_, CaO, and Fe_2_O_3_. The residues include polycyclic aromatics and other hydrocarbons [6]. From the perspective of composition, OSS has the roles of silicon source, carbon source, and metal ion source. After modification, OSS can be transformed into various valuable products, thus making the shale oil industry more sustainable. The zeolite-like materials were prepared from silica in oil shale minerals, demonstrating excellent adsorption properties for Cu(II), Pt(II), Zn(Ⅱ), and Cd(II) [7,8]. The hydrotalcite-like compound obtained from metal ions in oil shale minerals was applied to remove uranium from water. However, as far as we are aware, no studies have reported on producing high-value carbon materials from OSS.

Electricity demands have sky-rocketed and in order to conserve energy, the development of energy storage devices has become crucial for conserving energy. A variety of energy storage devices have emerged, including batteries and supercapacitors. Lithium-ion batteries remain the market leaders due to their high energy density, powerful capacity, mature technology, and complete industrial chains. The lithium–sulfur battery is now being hailed as a promising energy storage technology due to its low production cost and high theoretical capacity. In comparison with metal-ion charge carriers, NH_4_^+^ ions possess distinct characteristics, such as affordability, abundant resources, small hydrated ionic size, and light molar mass. These properties promote efficient ion diffusion in aqueous electrolytes [9,10].

Supercapacitors are an advanced energy storage technology that can achieve higher power density than batteries while also delivering higher energy density than traditional capacitors. Carbon materials are often used as electrode materials in supercapacitors due to their abundant porous structure, low cost, high electrical and thermal conductivities, and superior chemical stability. With the increasing demand for electrochemical energy storage devices, there are several challenges that need to be addressed in order to achieve high performance and longer service life, including reducing costs, improving safety, and addressing environmental protection concerns [11,12].

To date, increasing interest has focused on the conversion of waste to activated carbon materials for supercapacitors owing to their abundant resources and low prices. Furthermore, preparing activated carbon material from waste is also regarded as an effective method for waste treatment. Many wastes have been investigated for activated carbon starting materials, such as electronic, plastic, agricultural, and industrial wastes [13,14,15]. Among the numerous activation methods, chemical activation, in which the precursor is impregnated or mixed with an activating reagent and then heat treated under an inert atmosphere, is an essential method [16]. Playing an important role in the activation process, a variety of activating reagents such as K_2_CO_3_, NaOH, KOH, ZnCl_2_, FeCl_3_, H_2_SO_4_, and H_3_PO_4_ [17,18,19,20] have been proposed, and the advantages and disadvantages of each reagent have also been discussed. As an important activating reagent, KOH can produce micropores on the surface of materials at high temperatures to increase the surface area of materials [21]. When metal chlorides are used as activating reagents, the carbon content is usually increased by forming an aromatic graphite structure. Because of the dehydration of zinc chloride and the oxidation of organic compounds, the activated carbon shows a highly porous structure. In addition, microwave-assisted activation suggests significant advantages of internal heating and volumetric heating, quick transfer of energy, immediate start and blackout, and enhanced efficiency [22].

In this study, the main components of OSS (metal ion, silicon, and organic matter) were investigated for the purposes of environmental protection and high-value utilization of resources. The separation was carried out by microwave-assisted acid and alkali extraction. Activated carbon materials were prepared from the organic matter of OSS by different activation methods, and their electrochemical properties were investigated. The results demonstrated that the activated carbon is an effective electrode material for supercapacitors. The study not only develops a low-cost and effective way for preparing electrode materials, but also explores a new and appropriate strategy for OSS treatment, disposal, and utilization.

## 2. Results and Discussion

### 2.1. XRD Analyses

**Figure 1:**
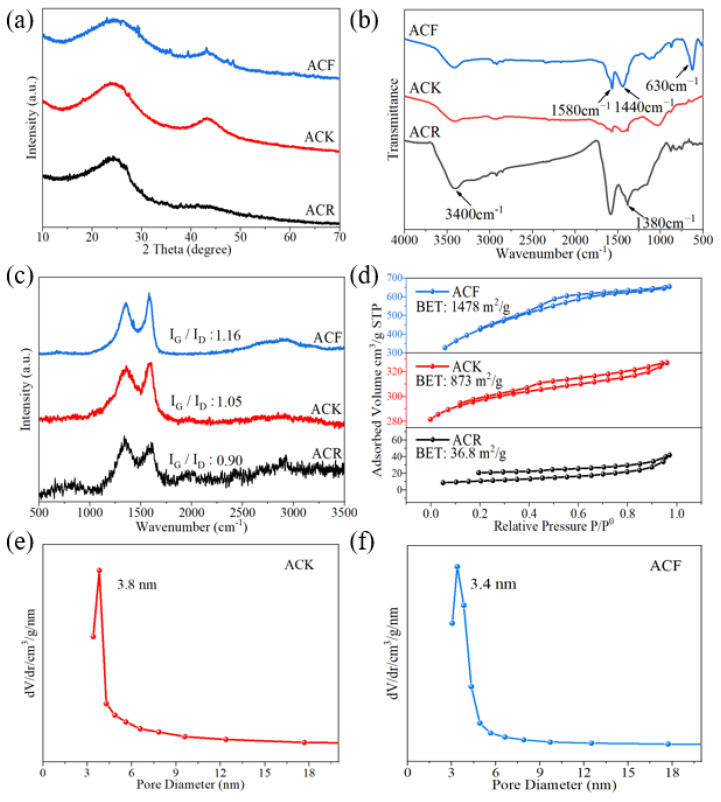
(**a**) XRD patterns, (**b**) FT-IR spectra, (**c**) Raman spectra, (**d**) N_2_ sorption isotherms of ACR, ACK, and ACF, (**e**) pore size distribution profile of ACK, (**f**) pore size distribution profile of ACF.

The difference in preparation methods will lead to variable structures of activated carbon. XRD analysis is an effective means to study the microcrystalline structure of activated carbon. As shown in Figure 1a, after being treated with alkaline solution, ACR shows significant diffraction peaks at approximately 24°; these correspond to (002) graphite structure crystal planes, indicating a certain degree of graphitization. A distinct diffraction peak appears around 43° corresponding to the (100) crystal planes of the graphite structure. Compared with ACR, the diffraction peak intensity of the (100) crystal plane of ACK obtained after further alkali treatment is significantly increased, which suggests that the carbon material structure is transformed from the original (002) crystal plane structure to (100) crystal plane structure under the action of high temperature and strong alkali, and the lamellar structure of graphite itself is increased.

Relevant studies used the Scherrer equation to evaluate the lateral size of the sp^2^-ordered crystallites from the full width at half maximum of the diffraction peaks. Lateral size increases with the increase in full width at half maximum [23]. In this paper, the full width at half maxima of ACR, ACK, and ACF increase successively, showing that the lateral size of the sp^2^-ordered crystallites increased from ACR to ACF. In addition, some sharp peaks can be observed in the XRD pattern of ACF, which were attributed to ZnFe_2_O_4_. ZnFe_2_O_4_ has attracted considerable attention for supercapacitors because of its high theoretical specific capacitance and distinct redox activity. However, intrinsically low electronic conductivity during the charge–discharge process severely hindered its applications in the supercapacitor. Studies have shown that ZnFe_2_O_4_ nanoparticles have been successfully added to cotton-derived active carbon fibers, which exhibit high specific capacitance and rate capability [24]. This indicates that the composite electrode material of ZnFe_2_O_4_ and activated carbon could also be prepared using the same preparation method as for ACF, which is very meaningful and needs further study.

### 2.2. FT-IR and Raman Analyses

FT-IR measurement was used to analyze the changes in the functional groups of the materials. As shown in Figure 1b, in the FT-IR spectrum of ACR, the characteristic bands at 3400 cm^−1^ were attributed to the stretching vibrational bands of adsorbed water and hydroxyl groups in activated carbon, the characteristic bands at 1630 cm^−1^ were attributed to the vibrational bands of carbonyl groups, and the absorption bands at 1390 cm^−1^ and 1585 cm^−1^ were attributed to the symmetric stretching bands and asymmetric stretching bands of carboxylate ions. The characteristic bands at 1180–1350 cm^−1^ were attributed to the absorption peaks of long-chain fatty acid salts, and the spectral band is wide, which indicated that the carbon chains attached to the carboxylic acids were not uniform in length [25]. Generally, when the material contains carboxyl, lactone, and other functional groups for which the pyrolysis products are CO_2_, it will hinder the charging process at the electrode interface and reduce the specific capacitance. When the material contains carbonyl and hydroxyl, for which the pyrolysis products are CO, the specific capacitance will increase. Therefore, ACR contains a large number of carboxyl groups, which is unfavorable for the electrochemical performance. Compared with ACR, ACK has been further treated with high temperature and strong alkali, and the functional groups on the surface are essentially removed. The high content of functional groups on the surface will increase the internal resistance of the material and reduce the life of the capacitor [26]. In the infrared spectrum of ACF, the characteristic bands at 1440 cm^−1^ and 1580 cm^−1^ correspond to the stretching vibration of the C=C bond in aromatic substances, and the characteristic bands at 630 cm^−1^ correspond to the absorption peaks generated by the deformation vibration of aromatic substances. This indicates that the activation by ZnCl_2_ and FeCl_3_ resulted in a significant increase in both the aromatic composition of the activated carbon and the graphitization of the material.

Irregular changes and defects in non-graphitized crystals form the D-band in the Raman spectrum. As shown in Figure 1c, the characteristic bands at 1342–1353 cm^−1^ (D-band, lattice breathing mode with A1g symmetry) are attributed to amorphous carbon with tiny grain size. The G-band is formed by the vibration of sp^2^ atoms in carbon rings or hexagonal crystals. The characteristic bands at 1590–1601 cm^−1^ (G-band) are attributed to graphitic carbon. It can be seen from the Raman spectra that, compared with ACR, the graphitization degree of ACK was enhanced after high temperature and alkali treatment. In the Raman spectrum of ACF, the intensity of the G-bands are enhanced, indicating an improved graphitization of the material. The relative intensity of the D-band and G-band signals is related to the degree of structural disorder and the lateral lattice size. The I(G)/I(D) ratios for ACR, ACK, and ACF were 0.90, 1.05, and 1.16, respectively, which indicates ACF has a more regular structure.

### 2.3. BET Surface Area Analyses

The specific surface area of activated carbon is considered to be the key factors affecting the electrochemical properties of the material. A large specific surface area will provide more active sites for electrolyte ions, thus enabling the carbon material to have higher double-layer capacitance. Theoretically, the larger the specific surface area, the higher the specific capacitance. When the specific surface area reaches 1600 m^2^/g, the specific capacitance reaches saturation and no longer increases with the increase in specific surface area [27,28]. As shown in Figure 1d, the specific surface area of ACK is only 873 m^2^/g, which indicates that high temperature and strong alkali treatment have no significant effect on the increasing in the specific surface area of the material. It is worth noting that the specific surface area of ACF is 1478 m^2^/g, indicating that ACF is more suitable as an electrode material than other materials in this study.

The pore size distribution of active carbon materials has an important effect on its specific electric capacity and the diffusion of electrolyte. In non-aqueous electrolyte systems, the 2–4 nm pores have an important effect on the electrolyte ion diffusion, which can not only effectively reduce the diffusion resistance of electrolyte, but also improve the utilization of specific surface area, thus enhancing the electrochemical performance of the material [29]. As shown in Figure 1e,f, the pore size distribution profiles of ACK and ACF, respectively, are 3.8 nm and 3.4 nm, which indicates that ACK and ACF are suitable as electrode materials.

### 2.4. Morphological Analysis

**Figure 2:**
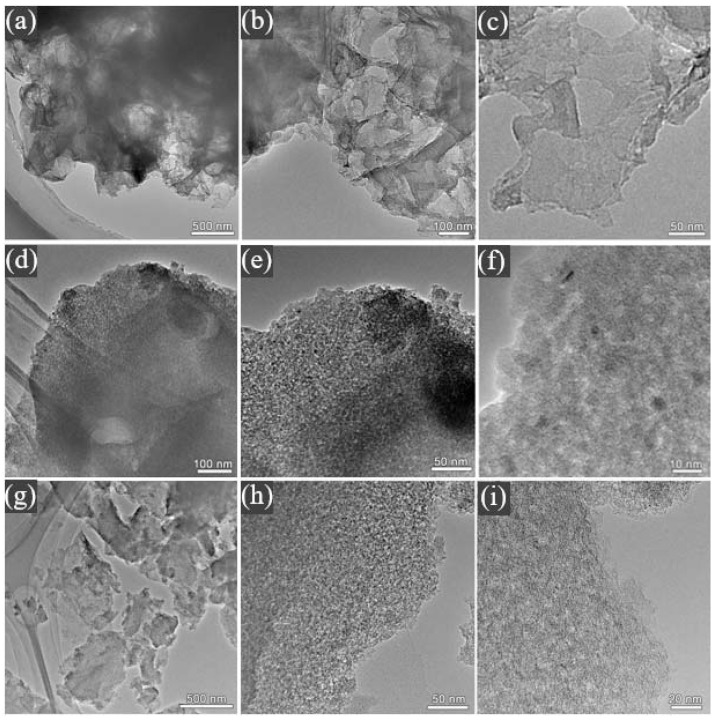
TEM micrographs of (**a**–**c**) ACR, (**d**–**f**) ACK, and (**g**–**i**) ACF.

As shown in Figure 2a–c, ACR has a spatial network structure with pores due to carbonaceous residues remaining mixed in with the minerals in OSS. Therefore, the inorganic components of the original oil shale semi-coke act as a “natural template”. The microstructure of ACR was further studied to find that the material exhibited a very thin lamellar structure, and the surface was smooth without pores, which was not conducive to the transfer of ionic liquid, and was unfavorable to the electrochemical performance of the material. As shown in Figure 2d–f, in ACK, the network structure of lamellar space disappeared, and was replaced by the formation of a connected hierarchical pore structure on its surface. These hierarchical pores provide more contact sites for electrolyte ions, which is conducive to improving the kinetic properties of the material [25]. From Figure 2g–i, it can be found that the thickness of lamellar ACF is significantly smaller than that of ACK, and there are more hierarchical pores on its surface, which not only increase the specific surface area of the material but also provide more contact sites for electrolyte ions.

### 2.5. Electrochemical Performance

**Figure 3:**
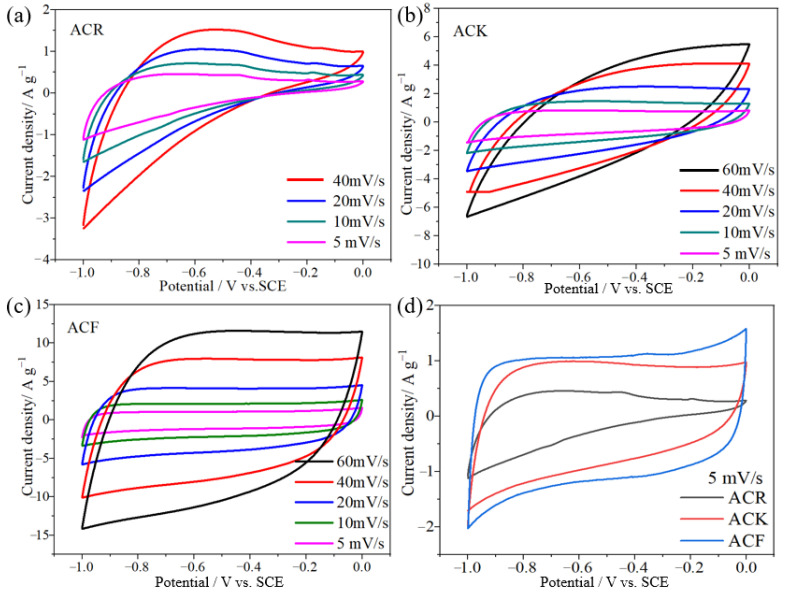
(**a**–**c**) The CV curves of ACR, ACK, and ACF electrode materials at different scanning speeds, respectively, (**d**) the CV curves of ACR, ACK, and ACF electrode materials at a scanning speed of 5 mV/s.

Figure 3a shows the CV curve of ACR at a scanning speed of 5–40 mV/s. The CV curves do not show the obvious rectangular shape ideal for carbon materials, but gradually approach a semicircle with the increase in scanning speed, which indicates that irreversible physical adsorption of electrode materials will occur with the increase in scanning speed, leading to the decrease in electrochemical performance. As shown in Figure 3b, the electrode prepared by ACK still maintains good symmetry when the scanning rate increases from 10 mV/s to 60 mV/s, indicating that ACK electrode material has better capacitance characteristics and rate capability after further activation. As the scanning rate of ACK material increases from 10 mV/s to 60 mV/s, the shape of its CV curve approaches an oval, which indicates that it is highly polarized. After further CV tests of ACF electrodes were performed at different scanning rates (Figure 3c), it was found that the rectangular shape of CV curve did not change significantly as the scanning rate increased from 10 mV/s to 60 mV/s, and still maintained good rectangular characteristics. This indicates that the unique structure of ACF electrode material gives it excellent capacitance characteristics and rate capability. Figure 3d shows the CV curves of several electrode materials at a scanning rate of 5 mV/s. Under this scanning rate, the CV curve of the ACF is a quasi-rectangular shape with good symmetry, which indicates that the ACF exhibits better double-layer capacitance characteristics than ACR and ACK.

**Figure 4:**
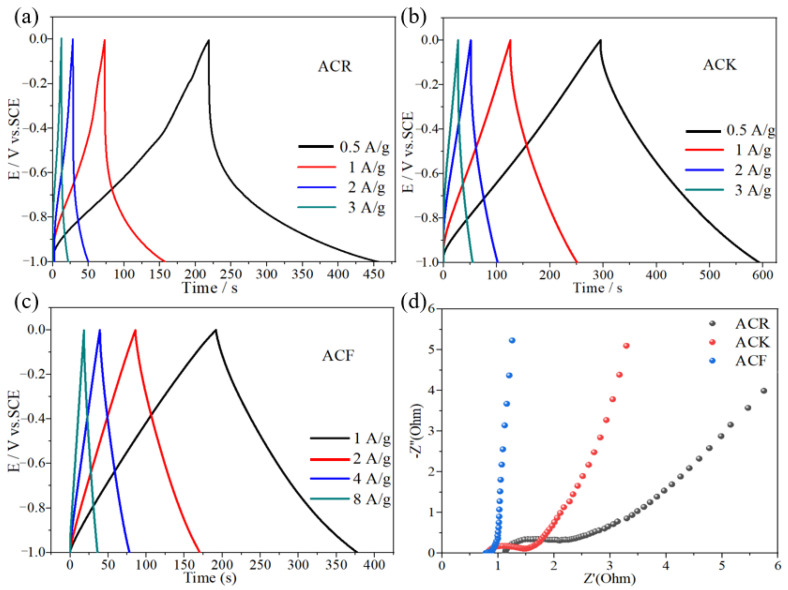
(**a**–**c**) The charge–discharge curves of ACR, ACK, and ACF electrode materials, respectively, (**d**) the electrochemical impedance spectroscopy of ACR, ACK, and ACF electrode materials.

The specific capacitance of ACR and ACK electrode materials at a current density of 1 A/g was 87 F/g and 129 F/g, respectively. After high temperature and strong alkali treatment, the specific capacitance of ACK electrode material was significantly improved, which was caused by the increase of pore structure and specific surface area. The porous structure is conducive to the diffusion of electrolyte ions in the electrode material, and the increase in specific surface area provides more adsorption sites for electrolyte ions. In addition, the charge–discharge curves of ACK electrode materials at different current densities show linear symmetry, which indicates that ACK electrode materials have good electrochemical reversibility. Rate capability refers to the ability of the electrode material itself to retain intrinsic capacitance during current charging and discharging. As shown in Figure 4a,b, when the current density is 0.5 A/g, 1 A/g, and 2 A/g, the specific capacitance of ACR electrode material is 118 F/g, 87 F/g, and 44 F/g, respectively, and the specific capacitance of ACK electrode material is 149 F/g, 129 F/g, and 100 F/g, respectively. This indicates that the rate capability of the ACK is significantly improved compared with the ACR. When the current density is 1 A/g, the specific capacitance of ACF and ACK electrode material is 185 F/g and 129 F/g, respectively. After the activation of FeCl_3_-ZnCl_2_ by high-temperature calcination, the specific capacity of the electrode material was significantly improved. This is mainly caused by the high specific surface area, which provides more adsorption sites for electrolyte ions. As shown in Figure 4c, the charge–discharge curves of ACF are linear and symmetrical at different current densities. When the current density is 1 A/g, 2 A/g, 4 A/g, and 8 A/g, the specific capacitance of ACF electrode material is 185 F/g, 169 F/g, 156 F/g, and 144 F/g, respectively. This indicates that ACF has excellent rate capability.

Electrochemical impedance spectroscopy (EIS) is an effective method for studying the kinetics of electrode processes [27]. The EIS diagrams of the ACF and ACK were tested at open-circuit voltage. As shown in Figure 4d, in the high-frequency region, the arc radius of ACR electrode material is the largest, indicating that the internal resistance of ACR electrode material is the largest, which indicates that in the electrode material, moderate pore size distribution can reduce the transfer resistance of electrolytes. In addition, compared to ACF, in the Nyquist diagram for ACK, the high-frequency region is a semi-circle, indicating that the ACF electrode has the smallest internal and charge transfer resistance. This is due to the high electrical conductivity of ACF electrode materials and the pore size which is conducive to the rapid electron-ion transfer. In the low-frequency region, the vertical degree between the linear part and the real axis of the three-electrode materials is ACR < ACK < ACF, indicating that the diffusion impedance of the three materials decreases successively, among which ACF has a lower diffusion impedance.

**Figure 5:**
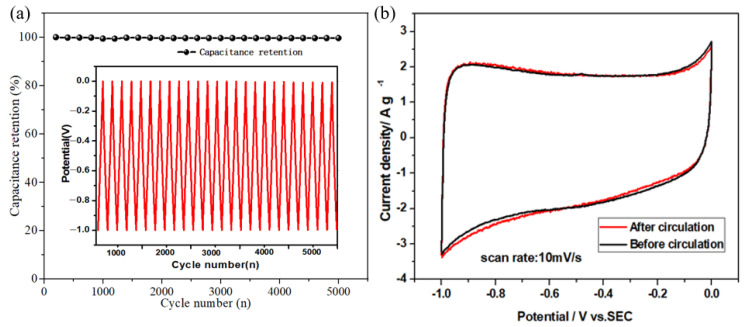
(**a**) Curves of Coulomb efficiency of ACF electrode material with the number of cycles, (**b**) CV curves before and after cycling.

The cycling performance of supercapacitors is one of the important factors to evaluate the practical application of supercapacitors. In this work, a three-electrode system was used, in which ACF electrode material was used as the working electrode to test its cycling performance in 6 mol/L KOH electrolyte. Figure 5a shows the curve of ACF electrode material after 5000 cycles of constant current charge/discharge at a current density of 10 mA/cm^2^. It can be found that after 5000 cycles of constant current charge/discharge, the specific capacitance of the electrode does not decay significantly, and its Coulombic efficiency remains at 99.5%, which indicates that ACF electrode material has good cycling performance.

### 2.6. Comparison with Other Activated Carbon

The electrochemical performance of some waste activated carbon reported in the literature is shown in Table 1. The results reveal that ACF can be a competitive material for supercapacitor electrode preparation.

## 3. Materials and Methods

### 3.1. Materials

Oil shale were collected from Dong Ning area in the northeast of China. The X-ray fluorescence chemical composition of OSS are SiO_2_ 65.90 wt%, Al_2_O_3_ 26.90 wt%, MgO 0.49 wt%, Fe_2_O_3_ 1.60 wt%, CaO 1.31 wt%, ZrO_2_ 0.025 wt%, SrO 0.019 wt%, Rb_2_O 0.0084 wt%, SO_3_ 0.39 wt%, K_2_O 2.05 wt%, and TiO_2_ 1.30 wt%. These samples were crushed and sieved to a particle sizes less than 2 mm; under nitrogen protection, the oil shale was calcined in a tubular furnace at 520 °C for 2 h and cooled to room temperature to obtain oil shale semi-coke, which was named OSS. All chemical reagents used in this experiment were of analytical grade and not purified.

### 3.2. Preparation of Activated Carbons (ACR, ACK, and ACF)

Microwave-assisted extraction was used to remove Al(III), Ca(II), Mg(II), and other metal ions from OSS by adding 2 mol/L HCl at a liquid:solid ratio of 10 mL/g, and the intermediate product OSS-1 was obtained by repeated extraction 4 times. Microwave-assisted extraction was used for further extraction silicate ions from OSS-1 by adding NaOH (30%) solution at a liquid:solid ratio of 10 mL/g, and the intermediate product OSS-2 was obtained by repeated extraction for 4 times. OSS-2 was then placed in an oven and dried at 60 °C for 24 h. The product obtained was named as ACR.

ACR and KOH were mixed in the mass ratio of 1:4, and the mixture was ground evenly and put into a tubular furnace under nitrogen condition, heated to 800 °C at a heating rate of 5 °C/min, and kept for 1 h. After the reaction, the product was cooled to room temperature, the product was washed with deionized water to neutral and placed in an oven, and dried at 60 °C for 24 h. The product obtained was named as ACK.

**Scheme 1:**
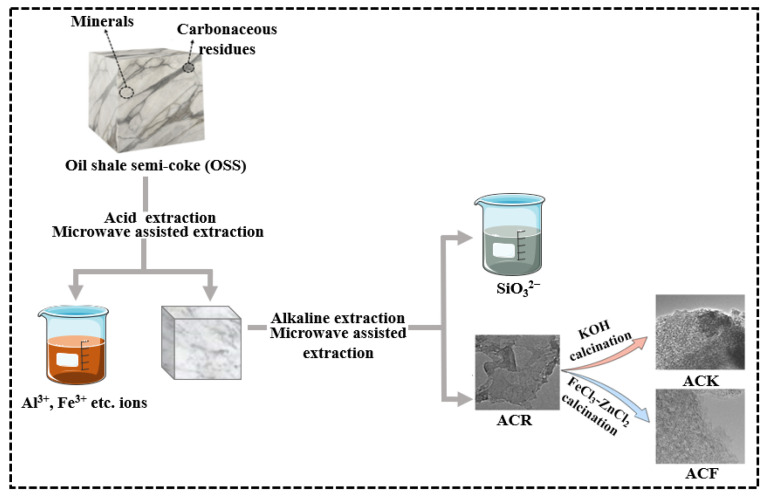
The synthesis of ACR, ACK, and ACF.

The precursor of active carbon was obtained by mixing 3 g ACR, 9 g ZnCl_2_, and 50 mL 3 mol/L FeCl_3_ solution, stirred for 2 h at 80 °C, placed in an oven, and dried at 120 °C for 12 h. The precursor was placed in a tubular furnace and heated to 800 °C at a heating rate of 5 °C/min under nitrogen condition for 1 h. After the reaction, the product was cooled to room temperature and washed with 2 mol/L HCl several times under ultrasonic conditions until it was not magnetic when tested by magnet. Subsequently, the product was washed with 2 mol/L HCl 3 times, placed in the oven, and dried at 60 °C for 24 h; the product was named as ACF. The synthesis process of ACR, ACK, and ACF is shown in Scheme 1. Optimal procedure of microwave digestion is shown in Table 2.

### 3.3. Electrochemical Measurement

The activated carbon materials (85% mass fraction of ACR, ACK, ACF), acetylene black (10%), and PTFE (5%) were mixed and coated on nickel foam (coating area was 1 cm × 1 cm) and dried at 60 °C for 12 h. The electrochemical tests were all carried out in the three-electrode system. The active carbon electrode was used as the working electrode, the counter electrode was 1 cm × 1 cm Pt sheet, the reference electrode was saturated calomel electrode (SCE), and the electrolyte was 6 M KOH solution. The operating voltage was −1.0 V to 0.0 V. The specific capacitance of the electrode was obtained by a constant-current charge–discharge curve. The calculation formula is as follows [44]:

C = I × Δt/(m × ΔV)(1)

where C is the specific capacitance (F/g), I is the discharge current (A), m is the mass of the active substance on the electrode (g), Δt is the discharge time (s), and ΔV is the operating voltage window (V).

### 3.4. Characterization Methods

Avatar 370 infrared spectrometer (Nicolet Company, Mountain, WI, USA) was used to analyze the changes in functional groups and chemical bonds of the samples. The test parameters were set as: scanning range 450–4000 cm^−1^, scanning times 32 times/min. Pretreatment method: The samples were ground and mixed with KBr at a mass ratio of 1:99 and pressed into tablets. LabRAM HR800 laser microscopic confocal Raman spectrometer (Horiba Jobin Yvon, Longjumeau, France) was used to detect the structure information of the samples. The composition and structure of the samples were analyzed by X’ Pert Pro multi-function X-ray diffractometer of Philip Company under the experimental conditions of Cu-Kα1 (λ = 0.15406 nm), the tube voltage is 40 kV, the tube current is 40 mA, and the scanning speed is 2°/min. Tecnai G20 high-resolution transmission electron microscope (FEI Company, Hillsboro, OR, USA) was used to characterize the micro-morphology of the samples. ASAP2010 physical adsorption analyzer (Micromeritics) was used to analyze the specific surface area and pore size distribution of the samples. The electrochemical properties of the samples were tested by using the CHI660D electrochemical workstation (Chenhua Company, Shanghai, China).

## 4. Conclusions

In this work, microwave-assisted alkali treatment and FeCl_3_-ZnCl_2_ mixed calcination methods were used to activate the carbon materials extracted from OSS. Raman, XRD, FT-IR, TEM, and nitrogen adsorption–desorption measurements were used to investigate the chemical components, structural, and morphological properties of activated carbon and the electrochemical properties of the products were investigated. It was shown that ACR does not have good electrochemical properties due to the low degree of graphitization, lack of pore structure on the surface, and low specific surface area. The electrochemical performance of ACK is improved compared to ACR due to the larger pore structure and specific surface area. Compared with ACR and ACK, the graphitization degree of ACF is further improved. The specific surface area of ACF is 1478 m^2^ g^−1^; when the current density is 1 A g^−1^, the specific capacitance is 185.0 F g^−1^. The conversion from OSS to activated carbon not only fabricates low-cost and high-performance electrode material, but also opens a new method for the comprehensive utilization of OSS.

## Figures and Tables

**Table 1:** The electrochemical performance of some waste activated carbon sources.

No	Waste	Surface Area (m^2^ g^−1^)	Specific Capacitance (F g^−1^)	References
1	Waste compact discs	1214	51	[30]
2	Rice husk	1768	171	[31]
3	Olive residue	1390	51	[32]
4	Wheat straw cellulosic foam	772	226.2	[33]
5	Cotton stalk	1481	114	[34]
6	Oil palm	1704	150	[35]
7	Bagasse	1892.4	142.1	[36]
8	Saw dust	1674	96.7	[37]
9	Mangosteen peel waste	1270	91	[38]
10	Cinnamon sticks	1540	53	[39]
11	CW-beech	1018	121.8	[40]
12	NCW@Fe_2_O_3_	642	603	[41]
13	NiCo_2_O_4_	—	1730	[42]
14	MnO@CNP-2	393.9	545	[43]
11	ACF	1478	185	This work

**Table 2:** Optimal procedure of microwave digestion.

Steps	Power/W	Heat-Up Time/min	Control Temperature/°C	Hold Time/min
1	700	8	100	3
2	1400	15	150	5
3	1400	15	200	15

## Data Availability

No new data were created.
